# A multidimensional learning curve analysis of totally laparoscopic ileostomy reversal using a single surgeon' s experience

**DOI:** 10.3389/fsurg.2023.1077472

**Published:** 2023-02-13

**Authors:** Zheng Xu, Yueyang Zhang, Hao Su, Xu Guan, Jianwei Liang, Qian Liu, Xishan Wang, Haitao Zhou

**Affiliations:** ^1^ Department of Colorectal Surgery, National Cancer Center/National Clinical Research Center for Cancer/Cancer Hospital, Chinese Academy of Medical Sciences and Peking Union Medical College, Beijing, China; ^2^ Department of Gastrointestinal Surgery, Key Laboratory of Carcinogenesis and Translational Research (Ministry of Education/Beijing), Peking University Cancer Hospital & Institute, Beijing, China

**Keywords:** colorectal cancer, ileostomy reversal, learning curve analysis, surgical oncology, totally laparoscopic surgery

## Abstract

**Purpose:**

Recently, totally laparoscopic ileostomy reversal (TLAP) has received increasing attention and exhibited promising short-term outcomes. The aim of this study was to detail the learning process of the TLAP technique.

**Methods:**

Based on our initial experience with TLAP from 2018, a total of 65 TLAP cases were enrolled. Demographics and perioperative parameters were assessed using cumulative sum (CUSUM), moving average, and risk-adjusted CUSUM (RA-CUSUM) analyses.

**Results:**

The overall mean operative time (OT) was 94 min and the median postoperative hospitalization period was 4 days, and there was an estimated 10.77% incidence rate of perioperative complications. Three unique phases of the learning curve were derived from CUSUM analysis, and the mean OT of phase I (1–24 cases) was 108.5 min, that of phase II (25–39 cases) was 92 min, and that of phase III (40–65 cases) was 80 min, respectively. There was no significant difference in perioperative complications between these 3 phases. Similarly, moving average analysis indicated that the operation time was reduced significantly after the 20th case and reached a steady state after the 36th case. Furthermore, complication-based CUSUM and RA-CUSUM analyses indicated an acceptable range of complication rates during the whole learning period.

**Conclusion:**

Our data demonstrated 3 distinct phases of the learning curve of TLAP. For an experienced surgeon, surgical competence in TLAP can be grasped at around 25 cases with satisfactory short-term outcomes.

## Introduction

A temporary loop ileostomy is frequently performed to avoid anastomotic leakage and protect the downstream anastomoses in colorectal cancer surgery (1). Subsequent reversal of the stoma might inevitably result in some complications, even for senior surgeons. According to the statistics, the reversal of ileostomy carries an estimated 17.3% morbidity rate and 0.4% mortality rate (2–4). With the evolution of minimally invasive techniques, laparoscopic-assisted reversal has been developed to reduce postoperative complications such as bowel obstruction and incisional hernia (5, 6). In addition, some initial explorations of laparoscopic reversal with intracorporeal anastomosis have been conducted (7–9). However, intracorporeal intestine reconstruction is relatively difficult and requires a learning process for inexperienced surgeons.

The learning curve can provide not only a visual representation of surgeon performance but also a quantitative estimation of surgical competency (10). Previous studies have analyzed the learning curve of intracorporeal anastomosis, suggesting that a plateau is reached after approximately 20–30 procedures (11–13). However, to our knowledge, the learning process of totally laparoscopic ileostomy reversal (TLAP) has not been previously investigated. In addition, most studies used operative time as the sole parameter to determine the learning curve and analyzed data using only one kind of statistical method, thus insufficiently representing the completion of surgical skill acquisition. In light of this, the present study was conducted to analyze the learning curve of TLAP based on operative time and perioperative complications using cumulative sum (CUSUM), moving average and risk-adjusted CUSUM (RA-CUSUM) analyses, aiming to show the safety and feasibility of this new technique.

## Methods

### Patients

In the second half of 2018, our group innovatively introduced the TLAP technique into ileostomy reversal. Since then, TLAP has been performed in >10 procedures/year by the same surgery team. From October 2018 to October 2021, a total of 65 consecutive patients were retrospectively enrolled. All patients with a history of laparoscopic colorectal cancer surgery underwent TLAP at the National Cancer Center/National Clinical Research Center for Cancer/Cancer Hospital, Chinese Academy of Medical Sciences and Peking Union Medical College.

In this study, any patients suited to undergo classic open reversal were regarded as potential candidates for TLAP. Eligible patients were those ≥18 years of age who received TLAP ≥3 months after former colorectal surgery or 8 weeks after postoperative chemotherapy/radiotherapy. Moreover, study participants also underwent both a colonoscope examination and enhanced computed tomography imaging of the thoracic, abdominal, and pelvic cavities to guarantee acceptable anastomotic stoma healing and exclude tumor recurrence or metastasis. Patients who underwent TLAP combined with additional procedures, such as additional intestine resection, anastomotic reconstruction, or parastomal hernia/abdominal wall repair, and those with other surgical contradictions for the traditional open ileostomy reversal were excluded from subsequent analysis. This study was in accordance with the Declaration of Helsinki and the informed contents were signed before the TLAP surgery. This research was also approved by the Ethical Committee of the Cancer Hospital (Institute), Chinese Academy of Medical Sciences, Beijing, People's Republic of China.

### Surgical team

The surgical team included a single experienced surgeon and 2 constant assistants throughout the study period. The participating senior surgeon has trained as an oncology surgeon for 15 years with extensive laparoscopic colorectal surgery experience (>100 procedures/year since 2015). Besides, TLAP was necessarily aided by a first assistant surgeon and a laparoscope holder. Both assistants were surgery residents and had completed 3 years of standardized training of residency after 2017. The primary duties of the first assistant surgeon included retraction and suction when necessary. All team members understood the details of TLAP technique, supported each other with effective methods, and would infuse their experience into their future performance.

### Surgical procedures

**Figure 1:**
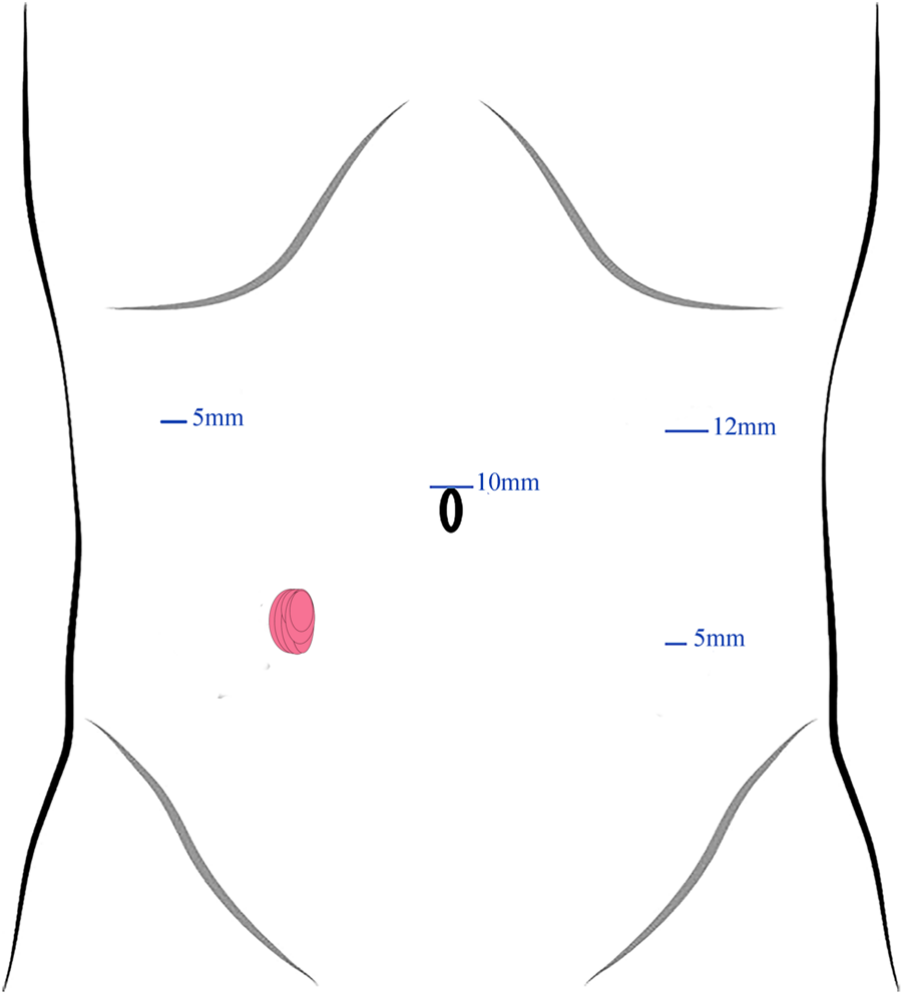
Trocar placement and the size of the trocars.

After general anesthesia, patients were placed in a supine lithotomy position and the previous stoma was closed in a one-layer continuous Lembert pattern wherein the needle exited the tissue within 1 mm of the stoma edge and engaged the submucosa with each bite. Here, a 4-port technique was employed for trocar placement (Figure 1). First, a 10-mm trocar was inserted upon the umbilicus as an observation port. Then, a 12-mm supraumbilical port was placed at the left anterior axillary line as a principal operating port. Next, a 5-mm operating port located at the left lower-quadrant McBurney's point was used for auxiliary operating. Another 5-mm port for assistant was located in the right anterior axillary line 10 cm superior to the stoma.

After the establishment of pneumoperitoneum, the lysis of adhesions around the stoma and dissection of the mesenteries were performed using an ultrasonic scalpel. A 60-mm endoscopic linear stapler (Johnson ECR60B) was subsequently used to transect the proximal and distal ileum for digestive tract reconstruction. First, a pair of 1-cm incisions located at the anti-mesenteric side of the proximal and distal intestines were made, respectively, and a side-to-side anastomosis was created with a 60-mm endoscopic linear cutter stapler (Johnson ECR60B). Then, the common opening of both intestines was closed by another linear cutter stapler and the mesenteric defect was closed by absorbable sutures routinely. Following examination of the anastomotic blood supply, the stoma remnant was removed and the incision was sutured conventionally.

### The learning curve analysis

In this study, operation time (OT) was regarded as a reflection of surgical competency. To explore the association between surgeon experience and OT, CUSUM and moving average analyses were completed. CUSUM analysis is an analytic technique employed in surgical procedures for the quantitative estimation and visualization of the learning curve (14). Briefly, the CUSUM is the total accumulated value of differences in OT between each data point and the mean OT of all data points. In the CUSUM analysis, all 65 cases were ordered chronologically from the earliest to the latest date of TLAP. For the first patient, the CUSUM_OT_ was the difference between the OT for the first patient and the mean OT for all cases. Similarly, the CUSUM_OT_ for the second patient was the difference between the second OT and the mean OT of all cases plus the CUSUM_OT_ for the first patient (15). This recursive process continued until the 65th patients was treated, and the results of all CUSUM_OT_ analyses were plotted graphically thereafter, revealing the trend of deviation from the mean OT. Of note, the inflection points indicated at each set of ≥3 consecutive negative values were used to divide patients into separate phases. A linear regression model was then fitted to match the CUSUM curve. In addition, we also used a moving average of 5 to eliminate individual variations and highlight the long-term trends of OT (16). Specifically, the moving average of the *i* cases was the mean value from the *i* cases to the *i* + 4 cases (17).

To analyze the learning curve from multiple dimensions, we designated each case as a success or failure. Conventionally, surgical failure was defined as conversion to open surgery. However, since there was no instance of conversion to open surgery, surgical failure was defined as any intraoperative or postoperative complications according to a previous report (18). Similar to the OT analysis, the CUSUM of complications was displayed graphically and showed the cumulative total of a mixture of increments with each surgery failure and decrements with each surgery success (19). Univariate and multivariate logistic analyses were then developed based on baseline variables (gender, age, body mass index et al*.*) to evaluate the potential confounders on surgical failure, respectively.

Furthermore, RA-CUSUM analysis was applied to depict the success or failure of the TLAP technique. First, baseline variables with *P *< 0.20 in the univariate association were considered for inclusion, and the predicted probabilities of each case were calculated according to the regression coefficients of the variables in the final multivariate regression model (20). Then, for each failure case, the RA-CUSUM value was incremented by (1-predicted probability of failure). In contrast, for each success, the value was decreased by the predicted probability of failure (21). Patients were again grouped into distinct phases according to the inflection points.

### Data collection and outcomes definition

The demographic and baseline variables included gender, age, body mass index (BMI), American Society of Anesthesiologists (ASA) score, duration after previous laparoscopic colorectal cancer surgery and comorbidities. Perioperative results included operation time, estimated blood loss, length of incision, time to ground activities and flatus passage, postoperative hospitalization, and perioperative complications. Estimated blood loss was the sum of the blood in the suction canister (the total volume after subtracting the amount of irrigation fluid) and the segment of increased weight of swabs during operation phase (1 ml of blood is about weighs 1 g) according to previous randomized controlled trails (22, 23). The time to ground activities and flatus was reported by patient. Postoperative hospitalization was defined as the number of nights from TALP to discharge. Perioperative complications were calculated within 30 days of surgery.

### Statistical analysis

The SPSS version 26.0 software program (SPSS Inc., Chicago, IL, USA) and Microsoft Office Excel were used for statistical analysis and data visualization, respectively. For quantitative variables with normal distribution as determined by the Shapiro-Wilk test, data are presented as mean ± standard derivation (SD) values and compared by One-way analysis of variance followed by Bonferroni's test. In contrast, data with skewed distribution are presented using median and interquartile range (IQR) values and compared by the Kruskal-Wallis test. For categorical variables, data are presented using numbers and percentages, and the chi-squared test or Fisher's exact test was applied to reveal group discrepancy. Polynomial regression models were selected according to a best-fitted model. A *P* value < 0.05 was considered to indicate a significant difference in all tests.

## Results

### Patient demographics and clinical profile

From 2018 to 2021, a total of 65 consecutive patients who underwent TLAP were enrolled in this study. The overall perioperative data are presented in Table 1. There were 43 male and 22 female patients treated with this innovative technique with a median age of 63 years. The mean BMI of the TLAP patients was 23.46 kg/m^2^. Most patients (87.69%) were classified as ASA class I or II cases, and the previous laparoscopic colorectal cancer surgery occurred a median of 9 months ago. Among comorbidities, hypertension was most common (21.54%), followed by diabetes mellitus, affecting 13.85% of enrolled patients. Other comorbidities included hyperthyroidism, coronary disease, and renal insufficiency.

**Table 1:** Patient demographics and clinical profile.

Characteristic	Value
**Gender**	
Male	33 (50.77)
Female	32 (49.23)
**Age, years**	63 (56–69)
**BMI, kg/m^2^**	23.46 ± 2.64
**ASA score**	
1–2	57 (87.69)
3–4	8 (12.31)
**Duration of ileostomy, mouths**	9 (7–10)
**Postoperative adjuvant therapy history**	33 (50.77)
**Comorbidities**	30 (46.15)
Hypertension	14 (21.54)
Diabetes mellitus	9 (13.85)
Hyperthyroidism	1 (1.54)
Coronary disease and hypertension	2 (3.08)
Hypertension and diabetes mellitus	3 (4.62)
Hypertension, coronary disease and renal insufficiency	1 (1.54)
**Operation time, min**	94 (80–105)
**Estimated blood loss, ml**	30 (20–30)
**Length of incision, cm**	6 (5–6)
**First ground activities, days**	1 (1–1)
**First flatus passage, days**	2 (2–3)
**Postoperative hospitalization, days**	4 (3–5)
**Intraoperative/postoperative complications**	7 (10.77)
Trocar site bleeding	1
Pyrexia	4
Incisional infection	2
Intra-abdominal infection	0
Incision fat liquefaction	0
Intestinal obstruction	0
Anastomotic bleeding	0

Data are presented as median (IQR), mean ± SD or *n* (%).

Intraoperative and postoperative data are also presented in Table 1. We found that the median operation time was 94 min, which was adopted as a crucial indicator for subsequent learning curve analyses. The estimated blood loss ranged from 10 ml–100 ml, with a median of 30 ml. The median incision length was 6 cm. Of note, in this study, the first ground activities (median = 1), first flatus passage (median = 2), and number of postoperative hospitalization days (median = 4) were used as reflections of postoperative recovery. Any complication during or after surgery was also estimated to assess the safety of the TLAP technique. In our series, a total of 7 patients suffered from intraoperative/postoperative complications (trocar site bleeding, *n* = 1; pyrexia, *n* = 4; incisional infection, *n* = 2).

### Learning curve analysis based on operation time

**Figure 2:**
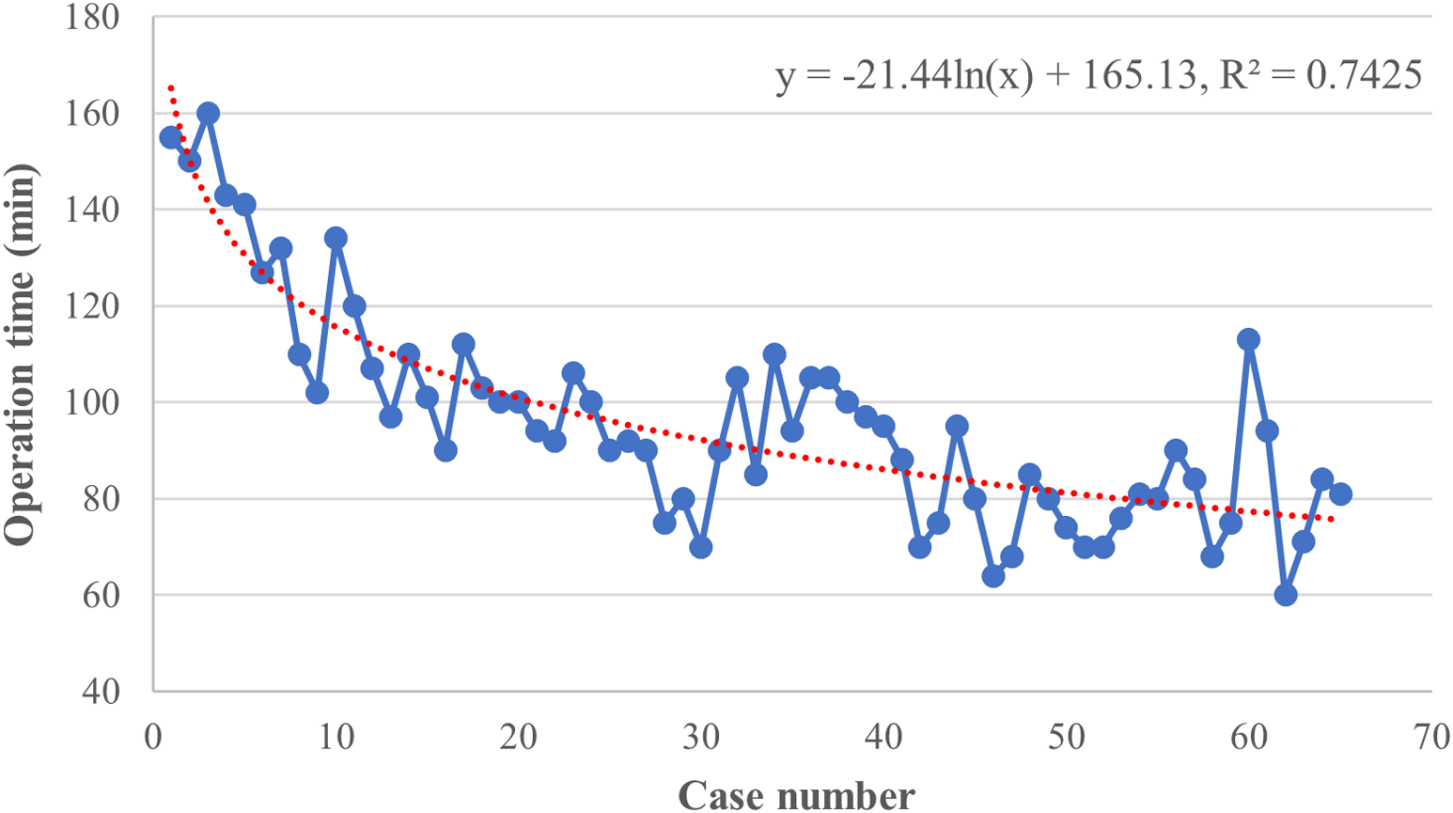
Graph of the raw operative time (OT) plotted against chronological case numbers (65 consecutive patients). The red dashed line represented the curve of best fit for the plot [*y* = −21.44 ln(*x*) + 165.13, *R*^2 ^= 0.7425].

**Figure 3:**
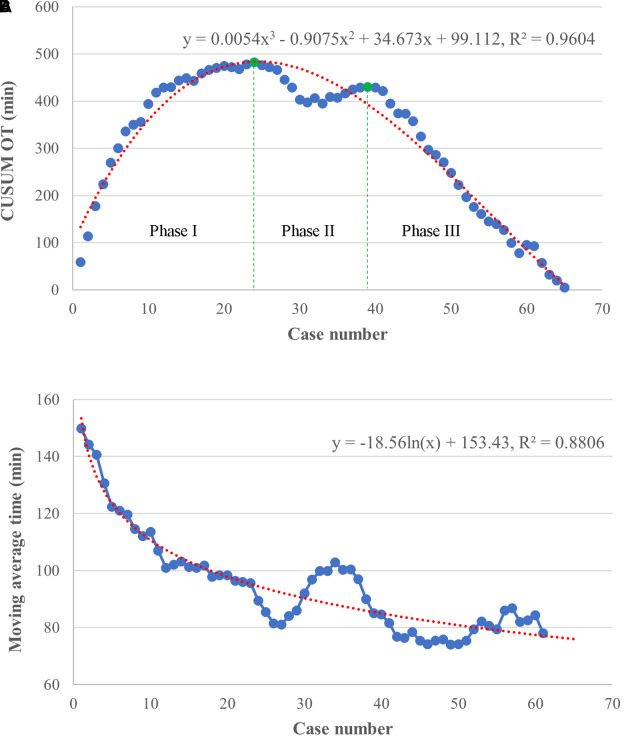
Results of cumulative sum (CUSUM) and moving average analyses based on operation time. (**A**) The CUSUM analysis of OT is best modeled by a red line as a third-order polynomial with equation *y* = 0.005*x*^3^–0.907*x*^2^ + 34.673*x* + 99.112, *R*² = 0.9604. The learning curve consisted of 3 unique phases (separated by green lines), as follows: an initial phase (case 1–24), a transition phase (case 25–39), and the proficient phase (case 40–65). (**B**) The moving average curve exhibited a gradual decline and was fitted with a logarithmic model of *y* = −18.56 ln(*x*) + 153.43, *R*² = 0.8806.

The raw operation time was plotted according to chronological case order and exhibited a tendency of steady reduction with the best-fitted logarithmic model [*y* = −21.44 ln(*x*) + 165.13, *R*^2 ^= 0.7425, *P *< 0.001], indicating a complex non-liner relationship between the OT and surgeon experience (Figure 2). CUSUM analysis was subsequently applied, and the mean operation time (96 min) was used as a critical reference. As shown in Figure 3A, the CUSUM of OT was best modeled as a third-order polynomial (*y* = 0.005*x*^3^–0.907*x*^2^ + 34.673*x* + 99.112, *R*² = 0.9604, *P *< 0.001), which showed a gradual upward slope until the 24th case, followed by small fluctuations between the 25th and 39th cases and a subsequent steep downward trend after the 39th case. Similarly, we determined that the OT decreased significantly after the 20th case and reached a steady state after the 36th case after fitting a logarithmic model of *y* = −18.56 ln(*x*) + 153.43 (*R*² = 0.8806, *P *< 0.001) in a moving average curve (Figure 3B).

**Figure 4:**
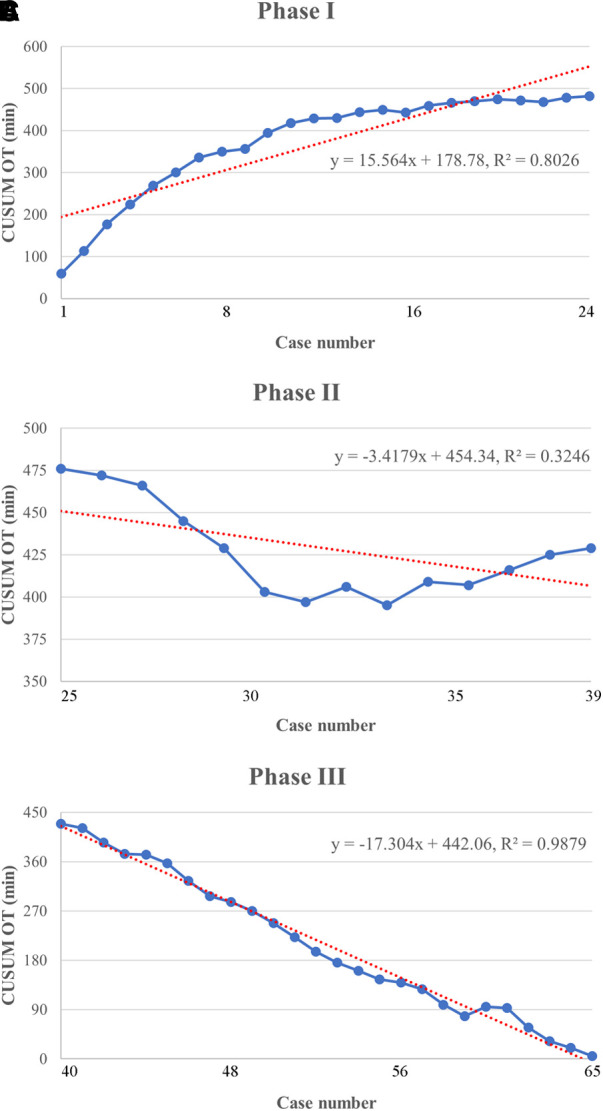
Lines of best fit for each phase of the CUSUM_OT_ learning curve. (**A**) Phase I of the CUSUM_OT_ learning curve represents the initial training phase (*y* = 15.564*x* + 178.78, *R*^2 ^= 0.8026). (**B**) Phase II of the CUSUM_OT_ learning curve represents the improvement phase (*y* = −3.4179*x* + 454.34, *R*^2 ^= 0.3246). (**C**) Phase III of the CUSUM_OT_ learning curve represents the mastery phase (*y* = − 17.304*x* + 442.06, *R*^2^ = 0.9879).

Based on the learning curve of the CUSUM of OT, we were able to separate the learning curve into the following 3 phases: phase I (an initial phase, including cases 1–24), phase II (a transition phase, including cases 25–39), and phase III (the proficient phase, including cases 40–65). Best-fitted lines for each phase were also acquired (Figure 4). The positive slope in phase I indicated a longer OT during the initial learning phase (*R*² = 0.8026, *P *< 0.001). However, a flat slope in phase II (*R*² = 0.3246, *P *= 0.027) revealed an increased degree of surgery competency in the transition phase. More importantly, the negative slope seen in phase III (*R*² = 0.9879, *P *< 0.001) confirmed the proficiency of the TLAP technique.

### Interphase comparisons between the learning phases

The interphase comparisons of patient characteristics are presented in Table 2. With regard to demographics, no statistical difference was found in gender (*P *= 0.742), age (*P *= 0.863), BMI (*P *= 0.067), ASA (*P *= 0.891), duration of ileostomy (*P *= 0.239), postoperative adjuvant therapy history (*P *= 0.535), or comorbidities (*P *= 0.187) among the initial, transition, and proficiency phases. Most notably, our results revealed that the OT between each phase was significantly different (108.5 min vs. 92 min vs. 80 min, *P *< 0.001). Phase I had the longest OT; meanwhile, a significant difference in OT was also revealed between phase II and III (*P *= 0.016). We additionally observed a significant downtrend in the number of postoperative hospitalization days (4 vs. 5 vs. 3 days, *P *< 0.001). In contrast, there was no significant difference in estimated blood loss (*P *= 0.988), surgical incision length (*P *= 0.798), time of first ground activities (*P *= 0.143), or time of first flatus passage (*P *= 0.663). Rates of intraoperative and postoperative complications between the 3 phases were not significantly different either (3 vs. 2 vs. 2, *P *= 0.778).

**Table 2:** Interphase comparisons of patient characteristics and perioperative outcomes.

	Phase I (*n* = 24)	Phase II (*n* = 15)	Phase III (*n* = 26)	*P* value
**Gender**				0.742
Male	13	7	13	
Female	11	8	13	
**Age, years**	64.5 (56.25–70)	58 (54–66)	61 (55.75–69)	0.863
**BMI, kg/m^2^**	23.03 ± 3.23	24.84 ± 2.07	23.07 ± 2.07	0.067
**ASA score**				0.891
1–2	22	13	22	
3–4	2	2	4	
**Duration of ileostomy, mouths**	8.5 (6–10)	9 (7–10)	10 (7.75–12)	0.239
**Postoperative adjuvant therapy history**	14	6	13	0.535
**Comorbidities**	10	10	10	0.187
**Operation time, min**	108.5 (100–133.5)	92 (85–105)	80 (70–85.75)	<0.001
**Estimated blood loss, mL**	30 (20–30)	30 (20–30)	30 (20–30)	0.988
**Length of incision, cm**	5.5 (5–6.75)	5 (5–6)	6 (5–6)	0.798
**First ground activities, days**	1 (1–1)	1 (1–2)	1 (1–1)	0.143
**First flatus passage, days**	2 (2–3)	2 (2–3)	2 (2–3)	0.663
**Postoperative hospitalization, days**	4 (3.25–5)	5 (4–5)	3 (3–3.25)	<0.001
**Perioperative complications**	3	2	2	0.778
Trocar site bleeding	0	1	0	
Pyrexia	1	1	2	
Incisional infection	2	0	0	

Data are presented as median (IQR), mean ± SD or *n* (%).

### Learning curve analysis based on complications

**Figure 5:**
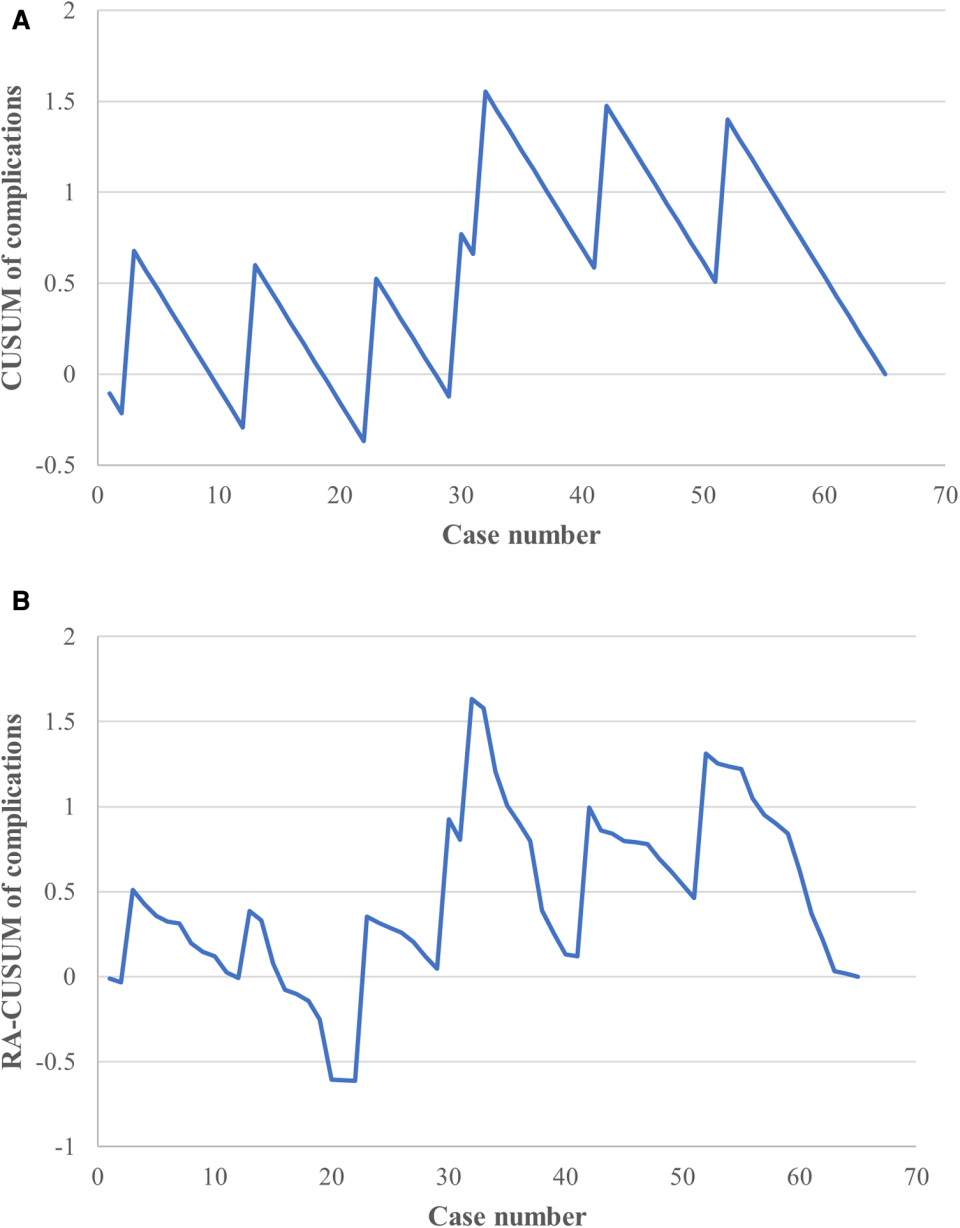
CUSUM and risk-adjusted CUSUM (RA-CUSUM) analysis for complications. (**A**) The CUSUM based on intraoperative/postoperative complications showed an acceptable fluctuation during the learning phase. (**B**) Small fluctuations were observed until the 22nd case, with a clear downward tendency seen after the 32nd case in the RA-CUSUM analysis.

To analyze the relationship between surgery experience and surgery success, CUSUM analysis was also performed. The CUSUM result based on intraoperative/postoperative complications showed a small fluctuation without a significant change between the zero line until approximately the 29th case, followed by an upward slope until the 32nd case and a subsequent downward slope thereafter (Figure 5A). To adjust for potential confounding effects of baseline covariables, univariable and multivariable logistic regression analyses were conducted (Table 3). The univariable analyses indicated that the gender (OR = 0.038, CI: 0.068–1.667; *P *= 0.183) and BMI (OR = 1.567, CI: 1.070–2.296; *P *= 0.021) were associated with a potential increased risk of surgery failure, with which age (*P *= 0.230), ASA score (*P *= 0.999), duration of ileostomy (*P *= 0.891), postoperative adjuvant therapy history (*P *= 0.722), and comorbidities (*P *= 0.540) were not significantly correlated.

**Table 3:** Logistic regression analysis of potential factors related to perioperative complications.

	Univariable analysis		Multivariable analysis	
	Odds ratio (95% CI)	*P* value	Odds ratio (95% CI)	*P* value
**Gender**	0.338 (0.068–1.667)	0.183	0.465 (0.084–2.489)	0.365
**Age**	0.957 (0.891–1.028)	0.230		
**BMI**	1.567 (1.070–2.296)	0.021	1.538 (1.044–2.265)	0.029
**ASA score**	0	0.999		
**Duration of ileostomy**	1.018 (0.789–1.313)	0.891		
**Postoperative adjuvant therapy history**	0.750 (0.154–3.652)	0.722		
**Comorbidities**	0.609 (0.125–2.970)	0.540		

In the multivariable analysis, BMI was the only factor independently associated with perioperative complications (OR = 1.538, CI: 1.044–2.265; *P *= 0.029). A further RA-CUSUM analysis was conducted based on the predicted odds ratio (Figure 5B); similar to the results of the CUSUM analysis, a small fluctuation was observed until the 22nd case and a downward tendency occurred after the 32nd case, suggesting an acceptable range with regard to perioperative complications during the learning period.

## Discussion

To date, some studies have reported on the initial exploration of totally laparoscopic ileostomy reversal, but no available data has shown the learning process of this technique. To the best of our knowledge, this is the first study to analyze the learning curve of TLAP. Using CUSUM and moving average analyses, we assessed the learning curve based on operation time and divided it into 3 distinct phases. Then, when we compared the perioperative parameters between these phases, we discovered a significant decrease in both the OT and hospitalization stay length when the level of TLAP performance was proficient. Furthermore, CUSUM and RA-CUSUM analyses illustrated an acceptable incidence of complications during the learning process. These results not only demonstrated a relatively short learning process of TLAP but also revealed its safety and feasibility, providing available proof for its future application.

Based on OT, we divided the learning process of TLAP into an initial phase, transition phase, and proficiency phase, respectively. According to CUSUM analysis, 25 cases were required for the initial exploration of TLAP, and another 14 cases were necessary to acquire proficiency. In contrast, 20 and 36 cases were required to complete the learning process, respectively, based on the moving average method. Despite limited studies of TLAP, some have explored the learning process of intracorporal intestinal anastomoses. In 2007, Torres et al*.* found that 21 cases were needed to achieve a satisfactory laparoscopic anastomoses time (12). In a recent study, the learning curve of laparoscopic right hemicolectomy with overlap anastomosis was decreased gradually and stabilized after 5 cases for experienced surgeons (24). Similar to our results, 18 cases were needed to gain increased competence based on the learning curve of right colectomy with intracorporeal anastomosis (25). Other studies investigating totally laparoscopic gastrectomy have suggested a required learning period of 27–29 cases (11, 13). Although these researches varied in their surgical approach, the key procedure was intracorporal anastomosis and digestive tract reconstruction. These learning curves in addition to the results of our present study may partially indicate a relatively short learning period is required for TLAP.

The learning curve for surgery complications showed an early peak, followed by a decreasing trend according to both the CUSUM and RA-CUSUM analyses. In other words, unlike the learning curve for operation time, the intraoperative/postoperative complications remained within an acceptable range from the early study stage onward (26). Admittedly, perioperative complications cannot be completely eliminated; however, their incidence was low in the initial, transition and postoperative periods, indicating the safety of the learning process and TLAP technique itself. Of note, the curve fluctuated until the 32nd case in both the CUSUM and RA-CUSUM analyses, which was attributed to trocar site bleeding during the operation in the 32nd case. According to the literature, ileostomy reversal carries an estimated 17.3% morbidity rate, which encompasses intestinal injury, small bowel obstruction, wound infection, and incisional hernia (27). In contrast, we observed an overall 10.77% rates of complications, and the majority were transient fever and incisional infection. Similarly, our previous study also reported a 10% incidence of postoperative complications associated with TLAP reversal in obese patients (9), whereas the open technique carried an increased incidence of incisional infection (26.5%). In summary, these results indicated the advantage of TLAP in reducing postoperative complications, which may be highlighted by further prospective and randomized multicenter studies.

We also identified a significant decrease in hospitalization stay length after the transition phase, suggesting a relationship between surgery experience and postoperative recovery. In addition, cumulative studies have revealed that the laparoscopy technique itself also contributes to a quick recovery. In a randomized controlled trial, the median length of hospital stay was significantly reduced after ileostomy closure with laparoscopy (5). Intracorporeal anastomosis also supported fast gastrointestinal function recovery in patients undergoing right hemicolectomy (28).

Notably, TLAP inevitably requires a surgical team that includes assistant surgeons. Although the auxiliary operators were inexperienced compared to the expert surgeon in this study, other studies have shown that a less-experienced assistant does not negatively affect perioperative outcomes (29, 30). Moreover, the learning curve also partly reflected the tacit team cooperation in TLAP. Therefore, the learning process of auxiliary surgeons was not presented independently in this study.

Admittedly, there are some limitations of this study that must be mentioned. First, this was a retrospective investigation with a small sample size in which baseline data were not fully balanced or randomized. Fortunately, further univariable and multivariable analyses showed no significant correlation between gender and complications. Second, a cost analysis assessment was not performed. However, we believe TLAP does not significantly increase hospitalization expenses based on a previous report (7). Third, the key step of TLAP, intracorporeal anastomosis, has been applied to patients undergoing right hemicolectomy in our group since 2016. As a result, enriched experience with laparoscopic colorectal surgery might be necessary to complete a safe and feasible TLAP. Lastly, current studies investigating TLAP are limited to small sample sizes, lacking adequate analysis of the learning process. In our institution, TLAP is not conducted by other surgeons either. As a result, the comparison of learning curves between different operators or studies might be difficult. More data should be made available from larger studies to illustrate the feasibility of TLAP thoroughly.

In conclusion, this study explored the learning process of TLAP from multidimensional perspectives. We not only differentiated 3 learning phases based on CUSUM, moving average and RA-CUSUM analyses but also found that reductions in operation time and hospitalization stay lengths and acceptable rates of perioperative complications were associated with mastery of the TLAP technique, providing reliable evidence of its potential for ileostomy reversal.

## Data Availability

The raw data supporting the conclusions of this article will be made available by the authors, without undue reservation.
